# Comparison of heads up three dimensional visualization system to conventional microscope in retinopathy of prematurity related tractional retinal detachment

**DOI:** 10.1038/s41598-021-01806-1

**Published:** 2021-11-16

**Authors:** Abdulrahman AlZaid, Wael A. Alsakran, Sulaiman M. Alsulaiman, Marco Mura

**Affiliations:** 1 Vitreoretinal Division, King Khaled Eye Specialist Hospital, P.O. Box 7191, Riyadh, 11462 Saudi Arabia; 2 Department of Translational Medicine, University of Ferrara, Ferrara, Italy; 3 Department of Ophthalmology, University of Illinois, Chicago, USA

**Keywords:** Retinal diseases, Retinopathy of prematurity

## Abstract

To report the outcomes, advantages and disadvantages of a heads-up three-dimensional (3D) visualization system compared to the conventional microscope in pediatric tractional retinal detachment (TRD) surgery secondary to advanced stage retinopathy of prematurity (ROP). Medical records of patients with ROP stage 4 or 5 who underwent surgery for tractional retinal detachment at King Khaled Eye Specialist Hospital between September 2017 and July 2019 were identified and reviewed. Eyes were divided into 2 groups, eyes that underwent surgery with a 3D heads-up platform (3D group) and eyes that underwent surgery with a conventional microscope (conventional group). Data were collected on neonatal history, visual acuity, intraoperative complications and success rates between groups.Eighteen eyes of 14 patients who underwent surgical repair of TRD related to ROP. Postoperative outcomes were compared between 10 eyes (7 patients) in the 3D group and 8 eyes (7 patients) in the conventional group There was no statistically significant difference in success rate between both groups (75% conventional group vs 70% 3D group). Partial or complete reattachment was achieved in 7 eyes in 3D group compared to 6 eyes in conventional group. Lower postmenstrual age at the time of the first surgery and presence of retinal breaks were associated with poorer surgical outcome. Heads up 3D visualization system is feasible in tractional retinal detachment related to ROP with similar success rate and no increased risk of complications when compared to conventional microscope. This system may be advantageous in advanced pediatric tractional retinal detachment surgeries.

## Introduction

The introduction of the three-dimensional viewing system (3D) to retinal surgery is an exciting addition to the surgical armamentarium. Previous studies have demonstrated the technical feasibility of 3D surgery^1,2^. This new viewing system offers many advantages including a more ergonomic “heads-up” posture for the surgeon, high image resolution at higher magnifications, potential enhanced visualization of different structures through the use of various filters (eg., to enhance vitreous visibility), lower endoillumination levels^3^, and a 3D viewing experience for the other staff in the operating room (e.g., doctors, nurses, students), which serves as an excellent teaching tool^1,2^. Despite fast adoption by many surgeons following market release, the feasibility, advantages and disadvantages of 3D visualization systems have not been assessed in advanced cases of pediatric ischemic vitreoretinopathies. This pathology is unique, as it usually requires anterior and posterior dissection which may limit surgeons’ adoption due to the fear of inadequate anterior segment viewing. In this report, we highlight safety and advantages of using a 3D visualization system for performing surgery in cases of pediatric tractional retinal detachment in retinopathy of prematurity (ROP). Additionally, we compare the surgical outcomes to the conventional microscope viewing system.

## Patients and methods

This interventional retrospective cohort study evaluated medical records of patients with ROP stage 4 or 5 who underwent surgery at King Khaled Eye Specialist Hospital, Riyadh, Saudi Arabia between September 2017 and July 2019 were identified and reviewed. Eyes were divided into 2 groups, eyes that underwent surgery with a 3D heads up platform (3D group) and eyes that underwent surgery with a conventional microscope (conventional group). Written informed consent was obtained from the parents for the surgical procedure. The study was approved by the Internal Review Board/Ethical Committee of King Khaled Eye Specialist Hospital, Riyadh, Saudi Arabia, and adhered to the tenets of the Declaration of Helsinki. Patient records were reviewed for demographic and ophthalmic data including gestational age, birth weight, postmenstrual age at the first surgery, and intraoperative and postoperative status of each eye. Preoperative and postoperative vision, when available, was noted. Surgical success was defined as complete or partial reattachment (posterior pole) of the retina. All surgeries were performed under general anesthesia by one surgeon (MM) who is an experienced user of different 3D visualization starting from 2015 and have a long experience in operating various complex retinal cases using this system. Eyes from the 3D group underwent surgery using the NGENUITY 3D system (Alcon Laboratories, Fort Worth, TX, USA) and eyes from conventional group underwent surgery using a conventional microscope. All patients underwent surgery with the same Leica M844 CT40 ceiling-mounted microscope (Leica Microsystems, Wetzlar, Germany), and with a 23, 25 and 27 gauge Constellation Vitrectomy platform (Alcon Laboratories, Fort Worth, TX, USA). The surgical technique consisted of a limbal approach lensectomy, total capsulectomy, followed by bimanual chandelier-assisted membrane dissection as previously reported by some of the authors^4^. Fluid -viscoelastic exchange was performed at the end of the procedure. Surgeon’s assessment of the advantages and drawbacks of the 3D visualization system was noted. Statistical comparison between groups was performed using the Mann–Whitney u test. *P* < 0.05 was considered statistically significant.

### Ethics approval

Approved by the Human Ethics Committee/Institutional Review Board of King Khaled Eye Specialist Hospital, Riyadh, Saudi Arabia, and adhered to the tenets of the Declaration of Helsinki.

## Results

Eighteen eyes of 14 infants, 7 males and 7 females, underwent surgical repair of tractional retinal detachment related to ROP were included in the study. Ten eyes (7 patients) underwent surgery in the 3D group and 8 eyes (7 patients) underwent surgery in the conventional group. Seventeen eyes had stage 5 ROP and 1 eye had stage 4B ROP. The eye with stage 4B disease underwent surgery with a conventional microscope. The mean gestational age of the study sample was 28.33 (SD +/− 1.75) weeks. The mean birth weight was 1028 (SD + /− 343) grams. The mean postmenstrual age at the time of first surgery was 76.3 (SD + /− 30.1) weeks. Bilateral retinal detachment was noted in 12 patients and 2 patients had unilateral retinal detachment. Both unilateral cases underwent surgery with a conventional microscope and the fellow eye had disease regression post-laser treatment. In 7 patients with bilateral retinal detachment, the fellow eye had stage 5 ROP but did not undergo surgery and in 1 patient the fellow eye underwent surgery at another hospital and had a detached retina that was not included in the study. Among all eyes, only 3 had received laser therapy prior to presentation (1 eye from the 3D group and 2 from the conventional group). Meanwhile, none of the eyes had received intravitreal anti-vascular endothelial growth factor or surgical treatment for retinal detachment. Twelve right eyes and 6 left eyes underwent surgery. Preoperative characteristics including gestational age, birth weight, postmenstrual age at the time of 1st surgery, presence of retrolental membrane, stage of ROP, configuration of detachment and preoperative vision were compared between both groups and shown in Table 1.

**Table 1:** Preoperative characteristics comparison between both groups.

	Conventional group	3D group	*P* value
Median (IQR) gestational age	28 (26.25–28.75) weeks	28 (27.75–29.75) weeks	0.192
Median (IQR) birth weight	800 (700–900) grams	1000 (900–1500) grams	0.081
Median (IQR) postmenstrual age at the time of 1^st^ surgery	93 (61.5–125.75) weeks	56 (46.75–90.50) weeks	0.075
Previous laser treatment	2 (25%)	1 (10%)	0.183
Retrolental membrane	3 (37.5%)	8 (80%)	0.145
**Stage of ROP**			
Stage 4B	1 (12.5%)	0	0.444
Stage 5	7 (87.5%)	10 (100%)	
**Configuration of detachment**			
Open Open	4 (50%)	1 (10%)	0.125
Open Close	0	3 (30%)	
Close Close	4 (50%)	6 (60%)	
**Preoperative vision**			
Follow light or object	2 (25%)	3 (30%)	> 0.999
No light perception	6 (75%)	7 (70%)	

*IQR* interquartile range, *ROP* retinopathy of prematurity.

Only 4 eyes with no light perception (NLP) vision underwent visual evoked potential testing and showed no response. Operative and postoperative characteristics including mean surgical duration, need for iridectomy, need for lensectomy, use of tamponade, number of procedures performed, vision at final follow up, retina attachment at final follow up, complications, surgical success and follow up period were compared between both groups and shown in Table 2.

**Table 2:** Operative and postoperative characteristics comparison between both groups.

	Conventional group	3D group	P value
Mean (SD) total surgical duration	118 (33) min	166 (72) min	0.07
Need for Iridectomy	0 (0%)	2 (20%)	0.47
Need for lensectomy	5 (62.5%)	10 (100%)	0.069
Use of silicon oil tamponade	1 (12.5%)	1 (10%)	> 0.999
Median (IQR) number of procedures preformed	1 (1–1)	1.5 (1–2)	0.096
**Vision at final follow up**			
Follow light or object	6 (75%)	8 (80%)	> 0.999
No light perception	2 (25%)	2 (20%)	
**Retina at final follow up**			
Partial or complete reattachment	6 (75%)	7 (70%)	> 0.999
Detached retina	2 (25%)	3 (30%)	
**Complications**			
Retinal break	2 (25%)	2 (20%)	> 0.999
Glaucoma	2 (25%)	1 (10%)	0.559
Surgical success	6 (75%)	7 (70%)	> 0.999
Mean (SD) follow up	20 (8.89) months	8.6 (2.87) months	0.007

*SD* standard deviation, *IQR* interquartile range.

There was a statistically significant improvement in vision from preoperative vision to vision at final follow up in both groups (*P* = 0.012). Variables that were significantly associated with surgical failure (complete retinal detachment) in both groups included a lower median postmenstrual age at the time of the first surgery (46 weeks vs. 89 weeks, *P* = 0.043) and presence of retinal breaks which was found to be 5.2 times higher risk of failure (*P* = 0.012). Variables that were not significantly associated with surgical failure included gestational age (*P* = 0.918), birth weight (*P* = 0.721), previous laser treatment (*P* > 0.999), presence of retrolental membrane (*P* = 0.596), configuration of detachment (*P* = 0.377) and need of lensectomy (*P* =  > 0.999).

## Discussion

This study presents the anatomical and visual outcomes of 10 eyes with stage 5 ROP that underwent surgical repair utilizing the NGENUITY 3D system for heads up vitreoretinal surgery in comparison to 8 eyes that underwent surgery with a conventional microscope. The outcomes of this study indicate that success rates with a 3D visualization system for surgery in advanced ROP were comparable to using a conventional microscope and to previous literature on ROP^5–13^.

Despite advances in neonatal care and ophthalmic care worldwide, stage 5 ROP continues to occur. In fact, 12% of eyes in the Early Treatment for Retinopathy of Prematurity study progressed to retinal detachment despite timely treatment^5^. Thus, surgical techniques for advanced ROP continue to evolve. Over time, surgeons shifted from open-sky techniques^6^ to closed vitrectomy techniques, now utilizing smaller gauge systems and various illumination techniques to achieve a less technically challenging surgery^4,7^.

Previous studies have reported widely divergent rates of anatomic success, ranging from 47 to 97%^5–13^. These studies included different stages of disease and utilized different surgical techniques, which may explain the variability between reports. In stage 5 ROP, partial (posterior pole) and total reattachment rates range between 22.5 and 48%^14–19^. Several factors affect the anatomical success rate including, but not limited to, the complexity of the funnel detachment and prior ablative therapy. In the current study, only 1 eye had an open-open funnel detachment in the 3D group and the remaining had a closed funnel component. In the conventional group, 4 eyes had open-open funnel detachment configuration and the remaining 4 eyes had a closed funnel component indicating easier configuration for surgical repair in this group. However, partial or complete reattachment rates were similar in both groups (70% of eyes in the 3D group and 75% in the conventional group).

Cusick et al. evaluated 183 eyes with stage 5 ROP and final visual outcome was 26% with NLP, 59% with LP, 10% had hand motion vision, and 4% were better than 5/200^14^. Seaber et al. evaluated 51 cases of stage 5 ROP and the postoperative vision was 29.4% with NLP, 21.6% with LP, 39.2% with hand motion vision, and 9.9% with ambulatory vision^20^. In our study final visual outcomes were similar between groups, with LP vision or better in 8 eyes (80%) in the 3D group and 6 eyes (75%) in the conventional group. However, maximum vision may be achieved with visual rehabilitation over several years.

The mean total surgical duration for each eye was 166 min in the 3D group compared to 118 min in the conventional group, although this difference was not statistically significant (*P* = *0.07*) as described in Table 2. It could be explained by the more complex configuration of detachment as shown in Table 1 which in turn required more than a single procedure for the 3D group as shown in Table 2.

The NGENUITY 3D system for vitreoretinal surgery consists of a 4,000-pixel display with an ultrafast, high-definition digital camera that allows for high-resolution, 3D viewing in the operating room. Studies have confirmed the efficacy and safety of this system in adult vitreoretinal surgery^21–23^. This new technology carries several advantages over a conventional microscope that were especially useful for ROP surgery. The main advantages noticed by our surgeon when working with NGENUITY 3D system compared to the conventional microscopes were enhanced depth perception and ability to operate under higher magnification with higher resolution. This was particularly helpful during membrane dissection in these complex detachments, as fine plane identification was facilitated with the system given the advantage of operating under high magnification with high resolution. Additionally, the 3D system contains different optical filters to change the color of the image, which aids in the recognition of the transparent vitreous in this young population. Image display lag was a concern with anterior segment work initially^24^, however, this was not perceived by our surgeon. The NGENUITY 3D camera can also digitally amplify the recorded signal to reduce endoillumination while maintaining sufficient visualization. Decreased endoillumination during long procedures such as stage 5 ROP surgery may reduce the risk for phototoxicity.

Another advantage is the 3D visualization for the assistant and all observers in the operating room. Since the learning curve is steep for ROP surgery, this feature could facilitate faster learning through direct teaching and high-quality recorded 3D videos. Additionally, experienced operating room staff can anticipate the surgeon’s needs as they can easily visualize the flow of surgery.

The limitations of this technology include the cost of the device, the initial learning curve, and possible difficulties during anterior segment portion of the surgery due to the adapting to the slight lag.

To our knowledge, this is the first report of the use of a 3D visualization system in tractional retinal detachments related to ROP. In our experience the NGENUITY 3D system seems to be as safe and effective as the conventional microscope while giving the surgeon the advantages of a digital viewing platform that increased image resolution even at higher magnifications. Limitations of the study include its retrospective nature, the small sample size and the relatively short follow up. However, this study shows the feasibility of using a 3D system for pediatric ischemic vitreoretinopathies and the potential advantages and limitations.

## Conclusion

Heads up 3D visualization system is feasible in tractional retinal detachment related to ROP with similar success rate and no increased risk of complications when compared to conventional microscope. 3D visualization system provides superior depth perception and magnification that allowed easier recognition of tissue planes. This system may be advantageous in advanced pediatric tractional retinal detachment surgeries.

## Data Availability

All data relevant to the study are included in the article.
